# Potential of amentoflavone with antiviral properties in COVID-19 treatment

**DOI:** 10.2478/abm-2021-0020

**Published:** 2021-08-20

**Authors:** Akhilesh Vikram Singh

**Affiliations:** School of Epidemiology and Public Health, Datta Meghe Institute of Medical Sciences, Sawangi (Meghe), Wardha, Maharashtra 442004, India

**Keywords:** amentoflavone, COVID-19, CYP450 inhibitor, flavonoid, SARS-CoV

## Abstract

Amentoflavone is one of the flavonoids that are known for their antiviral effects and many of them are predicted to have inhibitory effects against severe acute respiratory syndrome coronavirus (SARS-CoV) and Middle East respiratory syndrome Coronavirus (MERS-CoV) enzymes 3-chymotrypsin-like protease (3CLpro) and papain-like protease (PLpro). Amentoflavone is a biflavonoid found in the herbal extracts of St. John's wort (*Hypericum perforatum*), *Gingko biloba*, *Selaginella tamariscina, Torreya nucifera*, and many other plants. Its pharmacological actions have been listed as antiviral, antibacterial, antioxidant, anti-inflammatory, antidiabetic, antidepressant, and neuroprotective. Molecular docking studies have found that amentoflavone binds strongly to the active site of the main protease (M^pro^) of severe acute respiratory syndrome coronavirus-2 (SARS-CoV-2). As conventional antiviral medications are met with limited success against coronavirus disease-2019 (COVID-19) and vaccines are one of the only weapons against COVID-19 in the pharmaceutical armamentarium, traditional medicines are being considered for the forefront battle against COVID-19. Clinical studies with *Hypericum* and *Gingko* extract as additional or alternative drugs/supplements are registered. Here we review the potential of amentoflavone, an active agent in both *Hypericum* and *Gingko* extract as an adjunct therapy for COVID-19. Its anti-inflammatory, antioxidant, and sepsis preventive actions could provide protection against the “cytokine storm.” Compared with the herbal extracts, which induce cytochrome P450 (CYP) and uridine 5′-diphospho (UDP)-glucuronosyltransferases (UGT) activity producing a negative herb–drug interaction, amentoflavone is a potent inhibitor of CYP3A4, CYP2C9, and UGT. Further studies into the therapeutic potential of amentoflavone against the coronavirus infection are warranted.

Our more than 18-month long fight against the pandemic caused by the novel severe acute respiratory syndrome coronavirus 2 (SARS-CoV-2) has improved our understanding of some characteristics of the atypical respiratory disease coronavirus disease-2019 (COVID-19), but effective treatments to combat it are still far from sight. While vaccines have now passed phase 2 or 3 trials, traditional medicines are still being considered as possible treatments for COVID-19. The Chinese government has recommended traditional Chinese herbal medicine (TCM) as a therapeutic option for the treatment of COVID-19, and reports it to have >90% efficacy in fighting the Wuhan epidemic [1]. The Regional Expert Committee on Traditional Medicine for COVID-19 formed by the World Health Organization (WHO), the Africa Centre for Disease Control and Prevention, and the African Union Commission for Social Affairs have recently endorsed a protocol for phase III clinical trials of herbal medicine for COVID-19 [2]. As such, many natural products and herbal medicines known for their antiviral properties are being tested for effectiveness against the SARS-CoV-2. However, a drawback of these traditional medicines is that they are mixtures of different plant products with many active ingredients, and with no knowledge of their mechanism of action against new diseases like COVID-19. However, molecular docking studies in silico of active ingredients of various natural–herbal products have produced a list of molecules with probable anti-SARS-CoV-2 properties. These molecules can either block viral entry into cells via the angiotensin-converting enzyme 2 (ACE2) receptor and trans-membrane serine protease 2 (TMPRSS2), or inhibit the activity of 3-chymotrypsin-like protease (3CLpro) or RNA-dependent RNA polymerase (RdRp) by binding to their active sites. Of the various molecules tested in silico, the flavonoids top the hit list and show potential to bind to the catalytic site of SARSCoV-2 main protease (M^pro^) and thereby inhibit its replication in the host cell [3, 4]. Some of these flavonoids also show the potential to block the interaction of SARS-CoV S-protein with ACE2. Amentoflavone, a naturally occurring biflavonoid, tops the list of potential inhibitors of the SARS-CoV-2 protease [3,4,5,6,7]. A search on PubMed and PubFacts shows that amentoflavone has broad spectrum antiviral activity in addition to its anti-inflammatory and antioxidant effects, and is a naturally occurring human thrombin inhibitor [8, 9]. Here we critically analyze whether amentoflavone has the potential to be used as a therapeutic agent for COVID-19.

## Amentoflavone as an antiviral agent

Amentoflavone (C_30_H_18_O_10_; 4′,5,7-trihydroxyflavone)-(3′→8)-(4′,5,7-trihydroxyflavone; PubChem CID 5281600) is a biflavonoid of apigenin (3′,8″-bis-apigenin, didemethyl-ginkgetin). It has been isolated from nearly 120 plants used in traditional medicine by the Chinese, Indians, Africans, and Americans [8]. It is an active ingredient found in extracts from *Ginkgo biloba*, St John's wort (*Hypericum perforatum*), *Selaginella tamariscina, Torreya nucifera*, and many other plants, which have been used as dietary supplements in many countries. Amentoflavone has potent inhibitory activity against respiratory syncytial virus (RSV) at an IC_50_ of 5.5 μg/mL in vitro [10], significant antiviral activity against influenza A and B viruses, herpes simplex virus (HSV)-1 and HSV-2 with EC_50_ values of 17.9 μg/mL and 48.0 μg/mL, respectively, and against the reverse transcriptase of human immunodeficiency virus (HIV)-1 (IC_50_ 119 μM) [10]. Amentoflavone has inhibitory effects on drug resistant variants of HSV-1 [11] and hepatitis C Virus [12]. Amentoflavone inhibits coxsackievirus B3 viral replication by inhibiting fatty acid synthase (FAS) activity [13]. It reduced viral nuclear transportation through cofilin-mediated F-actin remodeling and inhibits immediate early gene expression in the HSV. Targeting the viral NS5A protein, amentoflavone inhibits cell entry, RNA replication, and polyprotein translation of hepatitis C Virus. It inhibits the NS5 RdRp of dengue virus at an IC_50_ = 1.3 μM and through molecular docking is predicted to be inhibitory against Zika virus NS3–NS2B protease [8, 14, 15].

**Figure 1:**
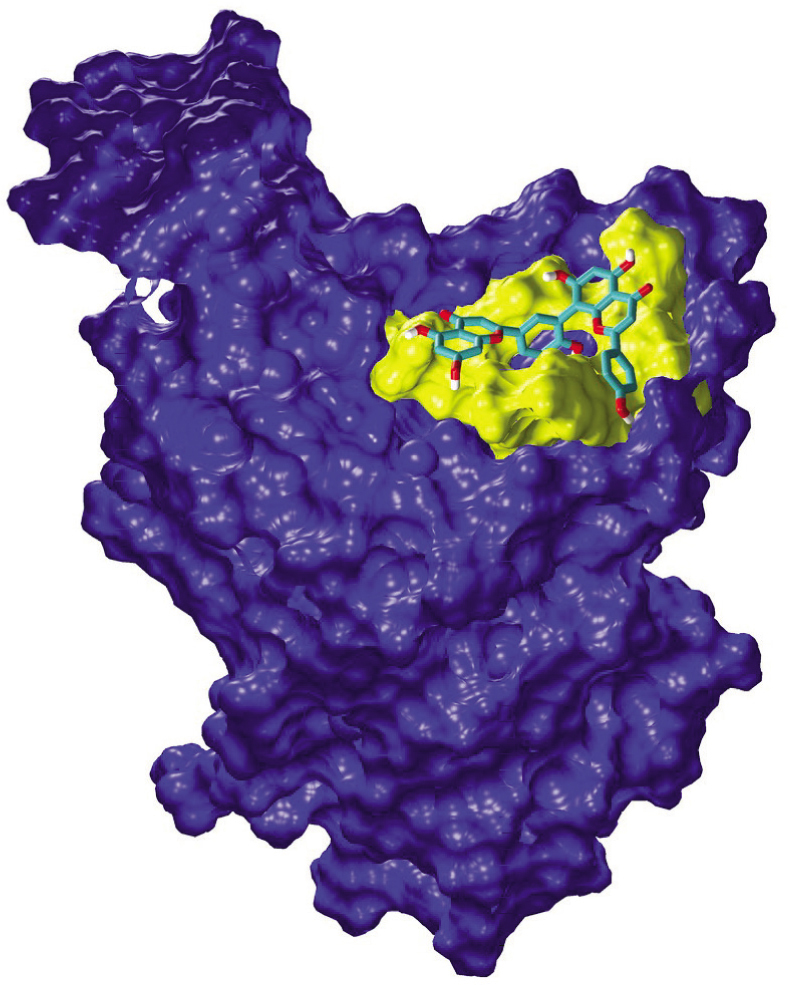
Molecular docking showing the binding of amentoflavone to the receptor binding domain of spike glycoprotein of SARS-CoV-2 (Research Collaboratory for Structural Bioinformatics Protein (RCSB) Data Base ID 6M17, chain E). Residues of the receptor binding domain interacting with amentoflavone are shown in yellow. Adapted from Miroshnychenko KV, Shestopalova A. Combined use of amentoflavone and ledipasvir could interfere with binding of spike glycoprotein of SARS-CoV-2 to ACE2: the results of molecular docking study. ChemRxiv. Preprint. doi: 10.26434/chemrxiv.12377870.v1, with kind permission of the authors. ACE2, angiotensin-converting enzyme 2; SARS-CoV-2, severe acute respiratory syndrome coronavirus 2.

Molecular docking studies predict amentoflavone to have potential to inhibit the SARS-CoV-2 by binding to its M^pro^ 3CLpro and thereby blocking the replication of the virus [3,4,5,6,7] (**Figure 1**). The M^pro^ is currently the most studied drug target of the SARS-CoV virus as its structure and substrate specificity are conserved amongst SARS-CoV, Middle East respiratory syndrome coronavirus (MERS-CoV), and SARS-CoV-2 [16]. Fortuitously, this viral protease shares no similarity with any human protease. Ryu et al. studying potential inhibitors of SARS-CoV responsible for severe acute respiratory syndrome (SARS) that spread from China to other Asian countries, North America, and Europe in 2002, reported amentoflavone as the most potent SARS-CoV 3CLpro inhibitor. Using a fluorogenic peptide (Dabcyl-KNSTLQSGLRKE-Edans), they showed that amentoflavone inhibited SARS-CoV 3CL^pro^ proteolytic activity in vitro at an IC_50_ = 8.3 μM and *K*_i_ = 13.8 ± 1.5 μM) [17]. The IC_50_ is lower than that for the antiviral flavones, such as its parent molecule apigenin (280.8 μM), and lute-olin (20.2 μM) and quercetin (280.8 μM), and is currently under clinical trials as a drug for the treatment of COVID-19 (ClinicalTrials.gov, NCT04377789, NCT04468139, NCT04536090). In addition, their study showed that amentoflavone binds strongly to the active site of 3CLpro having binding energy of −11.42 kcal/mol [17]. This has been supported by molecular dynamic docking studies [18, 19]. Amentoflavone has also been reported to bind to the receptor binding site of the spike glycoprotein of SARS-CoV-2 with a binding energy of −8.5 kcal/mol [20].

Such studies collectively emphasize the broad spectrum antiviral activity of amentoflavone, which can inhibit viral pathogenicity by targeting the viral proteins essential for its replication. As a functional FAS is essential for viral replication in general [13], the inhibitory effects of amentoflavone on FAS activity further makes it a promising antiviral therapeutic candidate. Studies with the fluorogenic peptide in vitro and docking studies in silico have shown a probable anti-SARSCoV effect of amentoflavone; however, to our knowledge, studies showing the inhibitory effects of amentoflavone on SARS-CoV 3CL^pro^ in any cellular system remain lacking.

## Can amentoflavone help combat COVID-19?

COVID-19 is regarded primarily as an atypical pulmonary disease characterized by severe pneumonia with acute respiratory distress syndrome (ARDS). Emerging data shows that this disease also leads to hyperinflammatory state resulting in other complications such as liver, kidney, and heart injury, and septic shock [21]. The cause of such complications is suggested to be due to “cytokine release syndrome” or a “cytokine storm” [22] and due to elevated serum cytokines (particularly interleukin (IL) 1β, IL-6, and tumor necrosis factor α (TNF-α)) levels, impaired interferon responses, and peripheral lymphopenia leading to lung injury [23]. Although the molecular mechanisms of coronavirus-induced pathogenesis are not entirely understood, emerging evidence suggests that, similar to the influenza A virus, SARS-CoV-2 infection possibly causes a rapid influx of inflammatory cells, which is followed by increased reactive oxygen species (ROS) production and elevated cytokine expression and release that leads to acute lung injury (ALI). Thus, it seems that inflammatory and immune response signaling along with ROS plays an important role in the pathogenic mechanism. SARS-CoV-2 also infects endothelial cells to cause endothelialitis that results in the activation of a coagulation cascade leading to vasculopathy, multisystem organ failure, and the hypercoagulable state observed in patients with severe cases of COVID-19 [23]. Thus, while considering traditional medicine, natural antiviral agents with added antioxidant and anti-inflammatory actions could prove effective in the treatment of COVID-19.

**Figure 2:**
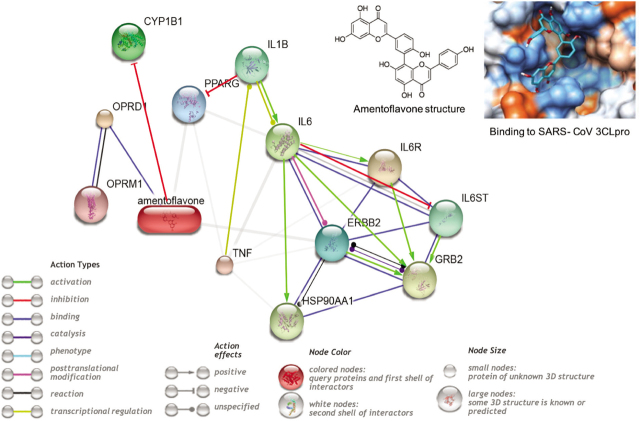
Interaction of amentoflavone with the inflammatory network and CYPs. Network drawn using STITCH showing chemical–protein interactions based on literature. In the network, nodes are proteins or chemicals and the edges represent the functional associations. The grey edges represent confidence with the line thickness indicating the strength of data support. Adapted from http://stitch.embl.de [25]. Inset shows the binding of amentoflavone to SARS-CoV 3CLpro. Adapted minimally and reprinted from [17] Bioorganic and Medicinal Chemistry, 18, Young Bae Ryu, Hyung Jae Jeong, Jang Hoon Kim, Young Min Kim, Ji-Young Park, Doman Kim, Thi Thanh Hanh Naguyen, Su-Jin Park, Jong Sun Chang, Ki Hun Park, Mun-Chual Rho, Woo Song Lee, Biflavonoids from *Torreya nucifera* displaying SARS-Co V 3CLpro inhibition, page 7944, Copyright (2010), with permission from Elsevier. The network shows the association of amentoflavone with CYPs and the inflammatory pathway to support the hypothesis for using amentoflavone as an adjunct therapy for COVID-19. 3CLpro, 3-chymotrypsin-like protease; COVID-19, Coronavirus disease-2019; CYP, cytochrome P450; ERBB2, v-erb-b2 erythroblastic leukemia viral oncogene homolog 2; GRB2, growth factor receptor-bound protein 2; HSP90AA1, heat shock protein 90α family class A member 1; IL1B, interleukin 1β; IL6, interleukin 6; IL6R, interleukin 6 receptor; IL6ST, interleukin 6 signal transducer; OPRD1, opioid receptor δ1; OPRM1, opioid receptor μ1; PPARG, peroxisome proliferator-activated receptor γ; SARS-CoV, severe acute respiratory syndrome coronavirus; TNF, tumor necrosis factor α.

Studies of the pharmacological functions of amentoflavone suggest it to be anti-inflammatory, antioxidative, and vasoprotective [8]. The scavenging effects with 2,2-diphenyl-1-picrylhydrazyl (DPPH), 2,2′-azino-bis(3-ethylbenzothiazoline-6-sulfonic acid (ABTS), superoxide, and hydroxyl radicals have shown it to have high antioxidant capacity (19.2%–75.5%) [24]. It significantly suppressed lipopolysaccharide induced nitric oxide (NO), ROS, malondialdehyde (MDA) in a rat astrocytoma cell line; NO, prostaglandin E-2 (PGE-2), the nuclear translocation of c-Fos in RAW 264.7 cells; and TNF-α in a human monocytic leukemia cell line, with no other apparent effects on the cells. It inhibited the oxidative burst of neutrophils and damage to human erythrocyte membranes induced in human peripheral blood mononuclear cells by phytohemagglutinin (PHA) by decreasing the levels of IL-6, TNF-α, IL-1β, and PGE2 [8] (**Figure 2**, [25, 26]). The anti-inflammatory effect of amentoflavone is due to its inhibition of the enzyme activity of extracellular signal-regulated kinase (ERK) that inactivates the ERK/c-Fos pathway suppressing the expression of genes for inflammatory mediators and decreased cytokine levels [27]. Amentoflavone can prevent sepsis induced ALI in rats by increasing the levels of superoxide dismutase and glutathione and decreasing thiobarbituric acid reactive substance levels [24, 28]. It protected lung tissue against cold stress-induced inflammation by binding to the complement component 3 (C3) and inhibiting the B cell receptor (BCR)/NF-κB and high mobility group box 1 protein (HMGB1) signaling [29]. Amentoflavone has also been suggested to be a naturally occurring inhibitor of human thrombin. Thrombin is the key protease of the coagulation pathway playing an important role in the regulation of blood coagulation cascade, and amentoflavone is a mixed-type inhibitor of human thrombin, with high binding potential (*K*_i_ = 8.06 μM) [9]. This property of amentoflavone could also aid against SARSCoV-2 pathogenicity caused by activation of the coagulation cascade. Amentoflavone can thus be considered as a promising therapeutic in the management of COVID-19.

## Characteristics of amentoflavone to consider

However, certain characteristics of amentoflavone should be noted. Like other flavonoids, the absolute oral bioavailability of amentoflavone is extremely low (0.06% ± 0.04%) and it undergoes rapid glucuronidation and sulfation in vivo [30]. The glucuronidation rate of amentoflavone is significantly higher than sulfation and it is the main metabolite in circulation. The area under curve (AUC_0−t_) of amentoflavone glucuronides (410.9 ± 62.2 ng/mL h) was significantly higher than the parent compound (194.5 ± 16.9 ng/mL h). Moreover, the antioxidant property of amentoflavone may remain unaltered following glucuronidation [30]. Studies suggest amentoflavone to be a potent inhibitor of cytochrome P450 (CYP) 3A4 and 2C9 [31, 32], cathepsin B [33], and also a broad spectrum inhibitor of uridine 5′-diphospho (UDP)-glucuronosyltransferases (UGTs) [34] (**Figure 2**). UGT1A1 and UGT1A3 are responsible for glucuronidation of amentoflavone and studies with microsomes in vitro in the presence of reduced nicotinamide adenine dinucleotide phosphate (NADPH) could not detect any oxidative metabolite of amentoflavone [30], suggesting that it is possibly not a substrate for CYP. At present, the effects of amentoflavone glucuronides on human UGTs or CYPs are not known.

As the uses of both conventional and traditional medications for COVID-19 are being explored, the possibility of herb–drug interactions should also be noted [35]. CYP monooxygenases and UGTs play a role in the metabolism and detoxification of xenobiotics and their excretion via urine or bile [36, 37]. This indicates a possible herb–drug interaction with amentoflavone. Clinical trials have been conducted with *G. biloba,* EGb761, and *H. perforatum* (St. John's wort) as both drug and dietary supplements to study their effects on depression, diabetes, cancer, acute coronary disease, inflammation, and Raynaud syndrome. Extracts of *G. biloba* contain only about 2% amentoflavone [38], while St. John's wort contains 0.01%–0.05% [39], and EGb761 does not contain amentoflavone. The herb–drug interactions noted with these extracts suggest them as inducers of CYPs and UGT. Any herbal extract contains many pharmacologically active ingredients and their effect is a combination of the function of each ingredient like the CYPs, and p-glycoprotein inducer activity of St. John's wort is attributed to hyperforin, hypericin, and quercetin [40]. The CYP and p-glycoprotein inducer activity of *G. biloba*/EGb 761 has been attributed to bilobalide, ginkgolide A, B, quercetin, and kaempferol [41]. Nevertheless, amentoflavone inhibits CYPs and UGT in vitro.

Being an inhibitor of both CYPs and UGT, the use of amentoflavone should be carefully reviewed to avoid the risk of toxicity due to increased bioavailability of the coad-ministered drugs. Administration of amentoflavone should also be calibrated against antithrombotic drugs like warfarin, rivaroxaban, or betrixaban to avoid bleeding. The possibility of administering amentoflavone as replacement of the conventional CYP 3A4 and 2C9, and UGT inhibitors, can also be considered. The currently approved antiviral drug remdesivir is an adenosine analog, which binds to the viral RdRp [42], and it can be administered with strong inhibitors of CYPs and UGTs [21]. Studies in vitro with HIV protease inhibitors like lopinavir, ritonavir, and darunavir suggest cleavage of 3CLpro and papain-like protease (PLpro) of SARS-CoV-2; however, they have been suggested to have poor selectivity in vivo as they have to be administered at extremely high doses [21, 42]. Thus, administration of anti-viral drugs such as remdesivir, lopinavir, and ritonavir with amentoflavone can result in increased efficacy. However, studies need to be conducted to ascertain the herb–drug interaction of amentoflavone and/or its main metabolites—the glucuronides.

## Conclusion

Although our understanding of the antiviral properties and mechanism of action of amentoflavone and its glucuronides is far from complete, the strong binding affinity of amentoflavone to SARS-CoV 3CLpro and spike glycoprotein predicts it be an effective natural therapeutic drug candidate against SARS-CoV-2. Its strong antioxidant property along with anti-inflammatory, thrombin inhibition, and protection against lung injury could aid in fight against the SARS-CoV-2 pathogenic complications like hyperinflammatory and hypercoagulable states that lead to ALI and multiorgan failure.

However, to provide a better understanding of its pharmacological effect against COVID-19 and using amentoflavone as an antiviral adjunct therapy against SARS-CoV-2, preclinical trials involving animal models need to be undertaken to confirm the antiviral activity of this biflavonoid, which, to our knowledge, has been shown only with assays in vitro. Although amentoflavone has been identified and isolated from many known medicinal plants and their herbal extracts that are used as dietary supplements, it should be noted that the content of amentoflavone in these extracts is very low. The highest content has been isolated from *Selaginella sp.* and that amounted to 65.31 mg (98% purity) from approximately 2.5 g of *S. tamariscina* [8]. A detailed study of the antiviral, anti-inflammatory, and antioxidant activity of either chemically or biologically synthesized amentoflavone and its metabolites is warranted. In addition, data on acute and chronic toxicological studies with amentoflavone in normal and diseased animal models are lacking.

With the advent of COVID-19 vaccines and rise of new and more virulent SARS-CoV-2 mutants, there is an urgent need to undertake pharmaceutical research of natural compounds that show strong binding affinity to the coronavirus in silico. As natural ingredients of *G. biloba* and St. John's wort, quercetin is already an investigational drug and rutin is suspected to be an active ingredient in TCMs that have been allegedly effective in COVID-19 prevention [43]; thus, ascertaining the effect of amentoflavone on SARS-CoV-2 infection will be of great interest. We hope this review will provide a stimulus for researchers to conduct detailed study of the antiviral and anti-inflammatory properties of amentoflavone as potential treatment for COVID-19 and proceed to preclinical and clinical trials with amentoflavone as adjunct therapy against this devastating disease.

## References

[1] Lee DYW, Li QY, Liu J, Efferth T (2021). Traditional Chinese herbal medicine at the forefront battle against COVID-19: Clinical experience and scientific basis. Phytomedicine.

[2] World Health Organization (2020). Expert panel endorses protocol for COVID-19 herbal medicine clinical trials [internet].

[3] Russo M, Moccia S, Spagnuolo C, Tedesco I, Russo GL (2020). Roles of flavonoids against coronavirus infection. Chem Biol Interact.

[4] Peterson L (2020). COVID-19 and flavonoids: in silico molecular dynamics docking to the active catalytic site of SARS-CoV and SARS-CoV-2 main protease. SSRN Electronic J.

[5] Jahan I, Onay A (2020). Potentials of plant-based substance to inhabit and probable cure for the COVID-19. Turkish J Biol.

[6] Orhan IE, Senol Deniz FS (2020). Natural products as potential leads against coronaviruses: could they be encouraging structural models against SARS-CoV-2?. Nat Products Bioprospect.

[7] Benarba B, Pandiella A (2020). Medicinal plants as sources of active molecules against COVID-19. Front Pharmacol.

[8] Yu S, Yan H, Zhang L, Shan M, Chen P, Ding A, Li SF (2017). A review on the phytochemistry, pharmacology, and pharmacokinetics of amentoflavone, a naturally-occurring biflavonoid. Molecules.

[9] Chen T-R, Wei L-H, Guan X-Q, Huang C, Liu Z-Y, Wang F-J (2019). Biflavones from *Ginkgo biloba* as inhibitors of human thrombin. Bioorg Chem.

[10] Ma S-C, But PP-H, Ooi VE-C, He Y-H, Lee SH-S, Lee S-F, Lin R-C (2001). Antiviral amentoflavone from *Selaginella sinensis*. Biol Pharm Bull.

[11] Li F, Song X, Su G, Wang Y, Wang Z, Jia J (2019). Amentoflavone inhibits HSV-1 and ACV-resistant strain infection by suppressing viral early infection. Viruses.

[12] Lee WP, Lan KL, Liao SX, Huang YH, Hou MC, Lan KH (2018). Inhibitory effects of amentoflavone and orobol on daclatasvir-induced resistance-associated variants of hepatitis C virus. Am J Chin Med.

[13] Wilsky S, Sobotta K, Wiesener N, Pilas J, Althof N, Munder T (2012). Inhibition of fatty acid synthase by amentoflavone reduces coxsackievirus B3 replication. Arch Virol.

[14] Coulerie P, Nour M, Maciuk A, Eydoux C, Guillemot J-C, Lebouvier N (2013). Structure-activity relationship study of biflavonoids on the Dengue virus polymerase DENV-NS5 RdRp. Planta Med.

[15] Bhargava S, Patel T, Gaikwad R, Patil UK, Gayen S (2019). Identification of structural requirements and prediction of inhibitory activity of natural flavonoids against Zika virus through molecular docking and Monte Carlo based QSAR Simulation. Nat Prod Res.

[16] Ullrich S, Nitsche C (2020). The SARS-CoV-2 main protease as drug target. Bioorganic Med Chem Lett.

[17] Ryu YB, Jeong HJ, Kim JH, Kim YM, Park JY, Kim D (2010). Biflavonoids from *Torreya nucifera* displaying SARS-CoV 3CLpro inhibition. Bioorganic Med Chem.

[18] Jo S, Kim S, Shin DH, Kim M-S (2020). Inhibition of SARS-CoV 3CL protease by flavonoids. J Enzyme Inhib Med Chem.

[19] Mishra A, Pathak Y, Kumar A, Mishra SK, Tripathi V (2021). Natural compounds as potential inhibitors of SARS-CoV-2 main protease: an *in-silico* study. Asian Pac J Trop Biomed.

[20] Miroshnychenko KV, Shestopalova A (2021). Combined use of the hepatitis C drugs and amentoflavone could interfere with binding of the spike glycoprotein of SARS-CoV-2 to ACE2: the results of a molecular simulation study. J Biomol Struct Dyn.

[21] National Institutes of Health (2021). COVID-19 Treatment Guidelines Panel. Coronavirus Disease 2019 (COVID-19) Treatment Guidelines [Internet].

[22] Iannaccone G, Scacciavillani R, Del Buono MG, Camilli M, Ronco C, Lavie CJ (2020). Weathering the cytokine storm in COVID-19: therapeutic implications. Cardiorenal Med.

[23] Torres Acosta MA, Singer BD (2020). Pathogenesis of COVID-19-induced ARDS: implications for an ageing population. Eur Respir J.

[24] Diniz LRL, Bezerra Filho CdSM, Fielding BC, de Sousa DP (2020). Natural antioxidants: a review of studies on human and animal coronavirus. Oxid Med Cell Longev.

[25] Szklarczyk D, Franceschini A, Wyder S, Forslund K, Heller D, Huerta-Cepas J (2015). STRING v10: protein–protein interaction networks, integrated over the tree of life. Nucleic Acids Res.

[26] Szklarczyk D, Santos A, von Mering C, Jensen LJ, Bork P, Kuhn M (2016). STITCH 5: Augmenting protein–chemical interaction networks with tissue and affinity data. Nucleic Acids Res.

[27] Oh J, Rho HS, Yang Y, Yoon JY, Lee J, Hong YD (2013). Extracellular signal-regulated kinase is a direct target of the anti-inflammatory compound amentoflavone derived from *Torreya nucifera*. Mediators Inflamm.

[28] Zong Y, Zhang H (2017). Amentoflavone prevents sepsis-associated acute lung injury through Nrf2-GCLc-mediated upregulation of glutathione. Acta Biochim Pol.

[29] Cai J, Zhao C, Du Y, Huang Y, Zhao Q (2019). Amentoflavone ameliorates cold stress-induced inflammation in lung by suppression of C3/BCR/NF-κB pathways. BMC Immunol.

[30] Gan L, Ma J, You G, Mai J, Wang Z, Yang R (2020). Glucuronidation and its effect on the bioactivity of amentoflavone, a biflavonoid from *Ginkgo biloba* leaves. J Pharm Pharmacol.

[31] Kimura Y, Ito H, Ohnishi R, Hatano T (2010). Inhibitory effects of polyphenols on human cytochrome P450 3A4 and 2C9 activity. Food Chem Toxicol.

[32] Park S-Y, Nguyen P-H, Kim G, Jang S-N, Lee G-H, Phuc NM (2020). Strong and selective inhibitory effects of the biflavonoid selamariscina A against CYP2C8 and CYP2C9 enzyme activities in human liver microsomes. Pharmaceutics.

[33] Pan X, Tan N, Zeng G, Zhang Y, Jia R (2005). Amentoflavone and its derivatives as novel natural inhibitors of human Cathepsin B. Bioorganic Med Chem.

[34] Lv X, Zhang J-B, Wang X-X, Hu W-Z, Shi Y-S, Liu S-W (2018). Amentoflavone is a potent broad-spectrum inhibitor of human UDP-glucuronosyltransferases. Chem Biol Interact.

[35] Ananchaisarp T, Rungruang S, Theerakulpisut S, Kamsakul P, Nilbupha N, Chansawangphop N (2021). Usage of herbal medicines among the elderly in a primary care unit in Hat Yai, Songkhla province, Thailand. Asian Biomed (Res Rev News).

[36] Stolbach A, Paziana K, Heverling H, Pham P (2015). A review of the toxicity of HIV medications II: interactions with drugs and complementary and alternative medicine products. J Med Toxicol.

[37] Borrelli F, Izzo AA (2009). Herb–drug interactions with St John's Wort (*Hypericum perforatum*): an update on clinical observations. AAPS J.

[38] Lobstein-Guth A, Briançon-Scheid F, Victoire C, Haag-Berrurier M, Anton R (1988). Isolation of amentoflavone from *Ginkgo biloba*. Planta Med.

[39] Nahrstedt A, Butterweck V (1997). Biologically active and other chemical constituents of the herb of *Hypericum perforatum L.*. Pharmacopsychiatry.

[40] Chrubasik-Hausmann S, Vlachojannis J, McLachlan AJ (2019). Understanding drug interactions with St John's wort (*Hypericum perforatum L*.): impact of hyperforin content. J Pharm Pharmacol.

[41] Deng Y, Bi H-C, Zhao L-Z, He F, Liu Y-Q, Yu J-J (2008). Induction of cytochrome P450s by terpene trilactones and flavonoids of the *Ginkgo biloba* extract EGb 761 in rats. Xenobiotica.

[42] Sanders JM, Monogue ML, Jodlowski TZ, Cutrell JB (2020). Pharmacologic treatments for coronavirus disease 2019 (COVID-19): a review. JAMA.

[43] Huynh T, Wang H, Cornell W, Luan B (2020). *In silico* exploration of repurposing and optimizing traditional Chinese medicine rutin for possibly inhibiting SARS-CoV-2's main protease. ChemRxiv. Preprint.

